# Effectiveness of Guselkumab for the Treatment of Moderate-to- Severe Psoriasis in Elderly Patients: A Retrospective, Real-World Multicenter Study in the Lazio Region

**DOI:** 10.3390/clinpract16070119

**Published:** 2026-06-26

**Authors:** Giuseppe P. A. Gemma, Nicoletta Bernardini, Francesca Paola Sasso, Giacomo Caldarola, Eleonora De Luca, Maria Elisabetta Greco, Viviana Lora, Annamaria Mazzotta, Emanuele Miraglia, Diego Orsini, Gianluca Pagnanelli, Antonio Giovanni Richetta, Nevena Skroza, Annunziata Dattola

**Affiliations:** 1 Dermatology Clinic, Department of Medical and Cardiovascular Sciences, Sapienza University of Rome, 00161 Rome, Italy; 2 Dermatology Unit, ASL Latina, Department of Medico-Surgical Sciences and Biotechnologies, Sapienza University of Rome, 04100 Latina, Italy; 3 UOC di Dermatologia, Dipartimento di Scienze Mediche e Chirurgiche, Fondazione Policlinico Universitario A. Gemelli—IRCCS, 00168 Rome, Italy; 4 Dermatologia, Dipartimento di Medicina e Chirurgia Traslazionale, Università Cattolica del Sacro Cuore, 00168 Rome, Italy; 5 Clinical Dermatology Unit, San Gallicano Dermatological Institute IRCCS, 00144 Rome, Italy; 6 Dermatology Unit, A.O. San Camillo Forlanini, 00151 Rome, Italy; 7 Clinic of Dermatology, San Sebastiano Hospital, Frascati, 00044 Rome, Italy; 8 Departmental Faculty of Medicine UniCamillus, “Saint Camillus International University of Health and Medical Sciences”, 00131 Rome, Italy; 9 Istituto Dermopatico Dell’immacolata IDI IRCCS, 00167 Rome, Italy

**Keywords:** psoriasis, guselkumab, elderly patients, real world, effectiveness

## Abstract

Background: Guselkumab, a selective interleukin-23 inhibitor, is effective in moderate-to-severe plaque psoriasis, but real-world long-term data in elderly patients remain limited. Objective: To evaluate the long-term effectiveness of guselkumab in elderly patients with moderate-to-severe psoriasis in a real-world setting. Methods: This retrospective, multicenter study included 102 patients aged 65 years or older treated with guselkumab across seven Italian centers. PASI scores and PASI 75/90/100 responses were assessed as observed cases at weeks 4, 16, 28, and 52, and annually up to 4 years, with last-observation-carried-forward and non-responder imputation as sensitivity analyses. Subgroups by biologic experience and BMI were analyzed. Results: Mean PASI decreased from 12.4 to 0.3 at week 52, remaining low through year 4 (*p* < 0.001). At week 52 and year 4, PASI 90 was achieved by 89.7% and 86.5%, and PASI 100 by 79.3% and 73.0%, respectively. Responses were comparable between biologic-naïve and biologic-experienced patients. Obese patients had higher baseline PASI; after adjustment for baseline severity, responses did not differ from non-obese patients. Conclusions: Guselkumab was associated with high and durable PASI improvement in elderly patients, regardless of prior biologic exposure or body weight. The retrospective design and absence of systematic safety data warrant cautious interpretation.

## 1. Introduction

Psoriasis is a chronic, immune-mediated inflammatory skin disease affecting approximately 2% of the global population. It is now recognised as a systemic condition, with up to 20–30% of patients developing psoriatic arthritis (PsA). Patients with moderate-to-severe psoriasis have an increased risk of metabolic syndrome, atherosclerotic cardiovascular disease, and depression, and the disease has a substantial negative impact on quality of life [1,2].

Psoriatic lesions result from the hyperproliferation and impaired differentiation of keratinocytes in the epidermis, which are triggered by immune mediators in the IL-23 and IL-17 pathway. This pathway is now recognised as one of the main causes of psoriasis pathogenesis [3,4,5].

Guselkumab is a biologic disease-modifying antirheumatic drug (bDMARD) that selectively targets interleukin-23 (IL-23) signalling by inhibiting its p19 subunit, preventing interaction with the IL-23 receptor. It has demonstrated effectiveness in improving both cutaneous symptoms of psoriasis and musculoskeletal manifestations of PsA [6,7,8,9].

The European Medicines Agency (EMA) recommends guselkumab as a therapeutic option for moderate-to-severe plaque psoriasis, administered at a dosage of 100 mg at week 0, week 4, and every 8 weeks thereafter. Although long-term safety and efficacy have been confirmed in pivotal clinical trials, real-world evidence in elderly patients remains limited. Despite its established efficacy in the general adult population, data specifically addressing the long-term effectiveness of guselkumab in elderly patients remain scarce, as this population is systematically underrepresented in pivotal clinical trials. This study aims to assess the long-term effectiveness of guselkumab in elderly patients with moderate-to-severe psoriasis in a real-world clinical setting [10,11,12,13].

## 2. Materials and Methods

This was a retrospective, observational study conducted across seven dermatology centres in the Lazio region, Italy. Because patients started guselkumab in different calendar years (2019–2025), the duration of available follow-up varied between patients, and not all subjects had reached the later time points at the time of analysis.

Patients aged ≥65 years with moderate-to-severe plaque psoriasis treated with guselkumab were enrolled.

No exclusion criteria were applied. Moderate-to-severe disease was defined as a Psoriasis Area and Severity Index (PASI) ≥ 10, or a lower PASI score in the presence of involvement of difficult-to-treat areas (face, palms/soles, genitalia, or nails).

Guselkumab was prescribed according to the Italian guidelines for the management of plaque psoriasis and the approved summary of product characteristics, in adult patients eligible for systemic treatment. The recommended dosage is 100 mg administered by subcutaneous injection at weeks 0 and 4, followed by 100 mg every 8 weeks thereafter [12,13].

This was a retrospective observational study based exclusively on irreversibly anonymized clinical data collected during routine clinical practice. Under applicable Italian regulations on observational studies individual informed consent was not required for the secondary use of irreversibly anonymized data, and patient consent was therefore waived [14]. The study was conducted in accordance with the principles of the Declaration of Helsinki (1975, revised in 2013). Data collection and handling complied with applicable data protection regulations, including the General Data Protection Regulation (GDPR).

Data Collection

Baseline demographic and clinical data included age, sex, disease duration, body mass index (BMI), comorbidities, prior exposure to biologic therapies, and involvement of difficult-to-treat areas (scalp/face, palms/soles, genitalia, and nails).

**Figure 1:**
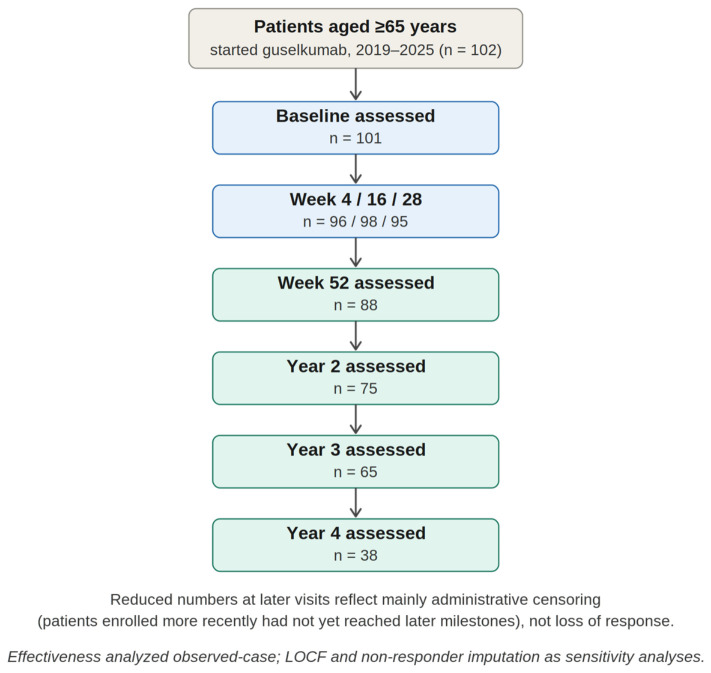
Patient flow through the study.

Clinical assessments were conducted at baseline and at scheduled timepoints: weeks 4, 16, 28, and 52, and annually at years 2, 3, and 4. The following outcomes were recorded: PASI score and PASI 75, PASI 90, and PASI 100 responses. Effectiveness was analysed primarily on an observed-case basis, using only patients with an available assessment at each timepoint; the number of patients assessed at each visit is reported (Figure 1). Reduced numbers at later visits reflected mainly administrative censoring—patients enrolled more recently had not yet reached the corresponding milestone—rather than treatment discontinuation. To test robustness against attrition, last-observation-carried-forward (LOCF) and non-responder imputation (NRI) were applied as sensitivity analyses.

Statistical Analysis

Continuous variables were summarised as mean ± standard deviation (SD) and median (interquartile range, IQR); categorical variables as absolute numbers and percentages. Normality was assessed with the Shapiro–Wilk test; as PASI scores were non-normally distributed at all timepoints, non-parametric methods were used. Changes in PASI over time were analysed with a linear mixed-effects model (random intercept per patient), which accommodates repeated measures and missing data, complemented by the Friedman test and Wilcoxon signed-rank tests versus baseline. Between-group comparisons (biologic-naïve vs. experienced; obese vs. non-obese) used the Mann–Whitney U test, with analysis of covariance (ANCOVA) adjusting for baseline PASI. Independent predictors of PASI 90 response were explored by multivariable logistic regression. Statistical significance was set at *p* < 0.05. Statistical analyses were performed using Python version 3.12 with the pandas, statsmodels, and lifelines libraries.

## 3. Results

The study population included 102 patients with moderate-to-severe psoriasis, comprising 52 males (51.0%) and 50 females (49.0%), with a mean age of 72.9 ± 6.5 years (range: 65–93 years). The mean body weight was 76.8 ± 13.1 kg, height 168.1 ± 8.6 cm, and BMI 27.1 ± 4.2 kg/m^2^, ranging from 18.3 to 43.0. The mean duration of psoriasis was 25.4 ± 17.0 years (range: 1–75), with a calculated mean age at disease onset of 47.5 ± 16.8 years. Prior exposure to biologic therapy was documented in 53 patients (52%), whereas 49 patients (48%) were biologic-naïve at the time of treatment initiation (Table 1).

Regarding disease phenotype, psoriatic arthritis was reported in 18 patients (17.6%), scalp involvement in 54 (52.9%), palmoplantar psoriasis in 17 (16.7%), genital involvement in 35 (34.3%), and nail involvement in 26 patients (25.5%). This heterogeneous clinical profile reflects a population with a long-standing, multifaceted disease burden, representative of real-world psoriatic disease.

Effectiveness

The Psoriasis Area and Severity Index (PASI) scores showed a significant reduction from baseline as early as week 4, with progressive improvement sustained throughout the follow-up period up to 4 years.

**Figure 2:**
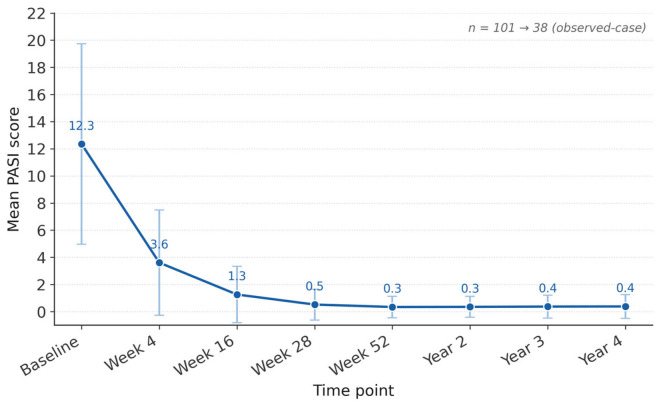
Evolution of mean PASI over time.

The mean PASI score decreased from 12.4 ± 7.4 at baseline to 3.6 ± 3.9 at week 4, 1.3 ± 2.1 at week 16, 0.5 ± 1.1 at week 28, and 0.3 ± 0.8 at week 52. This reduction was maintained at year 2 (0.3 ± 0.8), year 3 (0.4 ± 0.8), and year 4 (0.4 ± 0.9) (median 0.0 from week 28 onward; linear mixed-effects model −1.73 points/year, *p* < 0.001; all timepoints *p* < 0.001 vs. baseline) (Figure 2).

On an observed-case basis, PASI 75 was achieved by 58.3% at week 4, 85.7% at week 16, 95.8% at week 28 and 96.6% at week 52, remaining ≥95% through years 2–4.

PASI 90 was observed in 24.0% at week 4, 69.4% at week 16, 83.2% at week 28 and 89.7% at week 52, and was sustained (89.2%, 89.1%, and 86.5% at years 2, 3 and 4).

**Figure 3:**
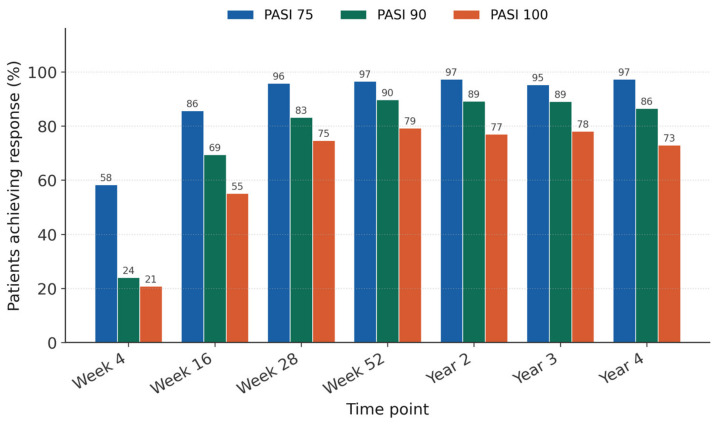
PASI 75/90/100 responses over time (observed case).

Complete clearance (PASI 100) was achieved by 20.8% at week 4, 55.1% at week 16, 74.7% at week 28 and 79.3% at week 52, and remained stable thereafter (77.0%, 78.1%, and 73.0% at years 2, 3 and 4) (Figure 3).

Impact of Previous Treatments

Of the 102 patients, 49 (48.0%) were biologic-naïve and 53 (52.0%) were biologic-experienced (27 with one, 16 with two, and 8 with three or more prior biologic lines). No significant difference in PASI was observed between the two groups at any time point (Mann–Whitney, all *p* > 0.05).

In biologic-naïve patients, the mean PASI score decreased from 13.2 ± 7.2 at baseline to 3.6 ± 3.7 at week 4, 1.3 ± 1.7 at week 16, 0.3 ± 0.8 at week 28, and 0.2 ± 0.6 at week 52, and was maintained at year 2 (0.3 ± 0.6), year 3 (0.2 ± 0.5), and year 4 (0.2 ± 0.5).

In biologic-experienced patients, the mean PASI improved from 11.6 ± 7.5 at baseline to 3.6 ± 4.1 at week 4, 1.3 ± 2.4 at week 16, 0.7 ± 1.4 at week 28, and 0.4 ± 0.9 at week 52, and persisted at year 2 (0.4 ± 0.9), year 3 (0.6 ± 1.0), and year 4 (0.5 ± 1.1).

Impact of body weight

**Figure 4:**
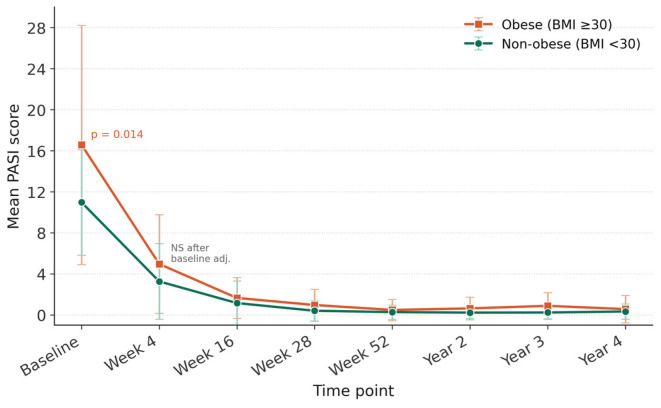
Mean PASI score over time by BMI category.

Obese patients (BMI ≥ 30, n = 22) had a significantly higher baseline PASI than non-obese patients (16.6 ± 11.7 vs. 11.0 ± 5.2; *p* = 0.014) (Figure 4). Although mean PASI at week 4 was numerically higher in obese patients, this difference was not statistically significant after adjustment for baseline PASI (ANCOVA, *p* = 0.352), indicating that the apparently slower early response was largely explained by higher baseline severity. From week 16 onward no significant differences were observed, and long-term outcomes were comparable between groups at year 2 (*p* = 0.088), year 3 (*p* = 0.016 in favour of non-obese), and year 4 (*p* = 0.979).

## 4. Discussion

In this multicenter real-world study with up to four years of follow-up, guselkumab showed sustained and clinically meaningful effectiveness in elderly patients (≥65 years) with moderate-to-severe plaque psoriasis, a population typically underrepresented in clinical trials. Reich et al. (2020) [15] assessed guselkumab in age-based subgroups; at week 24, patients aged ≥65 years achieved PASI 75, PASI 90, and PASI 100 in 87.8%, 75.6%, and 46.3%, respectively. In our cohort, at the closest timepoint (week 28), the corresponding observed-case responses were 95.8%, 83.2%, and 74.7%. Direct comparison is limited by the retrospective, uncontrolled design and by differences in patient selection and missing-data handling [15].

Blauvelt et al. (2022) [8] conducted a subgroup analysis by baseline age in patients from the ECLIPSE study; among guselkumab-treated patients aged ≥65 years (n = 54), 81.5% achieved PASI 90 and 53.7% achieved PASI 100 at week 48. Our week-52 observed-case rates (PASI 90 89.7%, PASI 100 79.3%) were in line with these figures, although direct comparison is constrained by the differences in study design noted above. Blauvelt et al. further reported durable responses over three years; the sustained low mean PASI scores in our cohort are consistent with a comparable pattern of durable disease control [8].

Subgroup analysis revealed no significant differences in treatment outcomes between biologic-naïve and biologic-experienced patients at any timepoint. Although biologic-naïve patients presented with slightly higher baseline PASI scores, both groups demonstrated significant and sustained improvement throughout the four-year follow-up period (*p* < 0.001 at all timepoints), with response rates converging from week 16 onwards. The proportions of patients achieving PASI 75, PASI 90, and PASI 100 were consistently high in both groups across all scheduled visits, indicating that prior biologic exposure did not adversely impact long-term treatment effectiveness. These findings support the use of guselkumab in biologic-experienced patients, including those with inadequate response, secondary failure, or intolerance to previous agents.

Similarly, obesity, a known negative predictor of response for certain biologic therapies, particularly TNF-α inhibitors, did not significantly affect long-term PASI outcomes in our cohort. Obese patients presented with higher baseline PASI scores (16.6 ± 11.7 vs. 11.0 ± 5.2; *p* = 0.014). Although mean PASI at week 4 was numerically higher in obese patients, this difference was not significant after adjustment for baseline severity (ANCOVA, *p* = 0.352), suggesting that the apparently slower early response was largely explained by greater baseline disease severity rather than by a true difference in drug response. Response rates remained comparable between groups throughout the four-year follow-up [16].

The study population was characterized by a markedly heterogeneous clinical profile, with a high prevalence of difficult-to-treat manifestations including psoriatic arthritis, nail, scalp, palmoplantar, and genital psoriasis (Table 1). These features are typically associated with reduced treatment satisfaction and impaired quality of life. Although overall response was high, site-specific outcomes for these phenotypes were not separately analysed, and effectiveness across the full spectrum of psoriatic phenotypes cannot be claimed on the basis of the present data [17,18,19].

Despite its strengths, this study has several limitations. First, its retrospective, uncontrolled design and the absence of a comparator group preclude causal inference. Second, patients were enrolled over different calendar years, so follow-up duration varied and later timepoints included fewer patients; although effectiveness was analysed on an observed-case basis with LOCF and non-responder imputation as sensitivity analyses, residual attrition bias cannot be entirely excluded. Third, safety data were not systematically collected, and no conclusions on tolerability can be drawn. Fourth, patient-reported outcomes such as the DLQI were not available, so clinical clearance may not fully capture patient-perceived benefit. Fifth, site-specific responses for difficult-to-treat areas and drug survival were not assessed. Finally, as the population was recruited from centres in the Lazio region, generalizability to other settings may be limited. Nevertheless, the extended follow-up of up to four years and the real-world multicenter design strengthen the external validity of these findings. Prospective studies with systematic safety capture and patient-reported outcomes are warranted.

## 5. Conclusions

In conclusion, this real-world multicenter study shows that guselkumab was associated with high and durable PASI improvement over a four-year follow-up in elderly patients with moderate-to-severe plaque psoriasis, regardless of prior biologic exposure or body weight. These findings are limited by the retrospective design and the absence of systematic safety data; prospective studies are needed to confirm long-term effectiveness and to characterise safety and patient-reported outcomes in this underrepresented population [20,21,22,23,24,25,26,27,28].

## Figures and Tables

**Table 1:** Baseline demographic and clinical characteristics of the study population.

Characteristic	All Patients (n = 102)
Demographics	
Age, years—mean ± SD (range)	72.9 ± 6.5 (65–93)
Sex, n (%)	
Male	52 (51.0)
Female	50 (49.0)
Weight, kg—mean ± SD	76.8 ± 13.1
Height, cm—mean ± SD	168.1 ± 8.6
BMI, kg/m^2^—mean ± SD (range)	27.1 ± 4.2 (18.3–43.0)
BMI category, n (%)	
Normal weight (<25)	31 (32.3)
Overweight (25–<30)	43 (44.8)
Obese class I (30–<35)	18 (18.8)
Obese class II–III (≥35)	4 (4.2)
Psoriasis history	
Disease duration, years—mean ± SD (range)	25.4 ± 17.0 (1–75)
Baseline PASI—mean ± SD	12.4 ± 7.4
Prior biologic exposure, n (%)	
Biologic-naïve	49 (48.0)
Biologic-experienced	53 (52.0)
1 prior line	27 (26.5)
2 prior lines	16 (15.7)
≥3 prior lines	8 (7.8)
Difficult-to-treat involvement, n (%)	
Scalp	54 (52.9)
Genital	35 (34.3)
Nail	26 (25.5)
Palmoplantar	17 (16.7)
Psoriatic arthritis	18 (17.6)
Comorbidities, n (%)	
Hypertension	44 (43.1)
Dyslipidemia	36 (35.3)
Diabetes mellitus	29 (28.4)
Previous malignancy	9 (8.8)
Thyroid disease	9 (8.8)
Latent tuberculosis infection	4 (3.9)
Hepatitis B	4 (3.9)
Hepatitis C	2 (2.0)
HIV	2 (2.0)
≥1 comorbidity	82 (80.4)
Comorbidity burden	
Charlson Comorbidity Index—mean ± SD	3.6 ± 1.5
Charlson Comorbidity Index—median (IQR)	3 (3–4)

## Data Availability

The datasets generated and/or analysed during the current study are not publicly available due to privacy and ethical restrictions related to patient data protection (GDPR). However, anonymised data may be available from the corresponding author on reasonable request.

## Abbreviations

The following abbreviations are used in this manuscript:

PASI	Psoriasis Area and Severity Index
BMI	Body Mass Index
PsA	Psoriatic Arthritis
EMA	European Medicines Agency
bDMARD	biologic Disease-Modifying Anti-Rheumatic Drug
LOCF	Last Observation Carried Forward
IL	Interleukin

## References

[1] Nestle F.O., Kaplan D.H., Barker J. (2009). Psoriasis. N. Engl. J. Med..

[2] Lebwohl M.G., Bachelez H., Barker J., Girolomoni G., Kavanaugh A., Langley R.G., Paul C.F., Puig L., Reich K., van de Kerkhof P.C. (2014). Patient perspectives in the management of psoriasis: Results from the population-based Multinational Assessment of Psoriasis and Psoriatic Arthritis Survey. J. Am. Acad. Dermatol..

[3] Hawkes J.E., Yan B.Y., Chan T.C., Krueger J.G. (2018). Discovery of the IL-23/IL-17 Signaling Pathway and the Treatment of Psoriasis. J. Immunol..

[4] Brembilla N.C., Boehncke W.H. (2023). Revisiting the interleukin 17 family of cytokines in psoriasis: Pathogenesis and potential targets for innovative therapies. Front. Immunol..

[5] Cai Y., Fleming C., Yan J. (2012). New insights of T cells in the pathogenesis of psoriasis. Cell Mol. Immunol..

[6] Chiricozzi A., Costanzo A., Fargnoli M.C., Malagoli P., Piaserico S., Amerio P., Argenziano G., Balato N., Bardazzi F., Bianchi L. (2021). Guselkumab: An anti-IL-23 antibody for the treatment of moderate-to-severe plaque psoriasis. Eur. J. Dermatol..

[7] Dattola A., Bernardini N., Anedda J., Atzori L., Bonifati C., Bruni P.L., Giordano D., Graceffa D., Molinelli E., Moretta G. (2025). Real-world analysis of IL-23 inhibitors in patients with moderate-to-severe psoriasis and early musculoskeletal symptoms. Drugs Context.

[8] Blauvelt A., Armstrong A.W., Langley R.G., Gebauer K., Thaçi D., Bagel J., Guenther L.C., Paul C., Randazzo B., Flavin S. (2022). Efficacy of guselkumab versus secukinumab in subpopulations of patients with moderate-to-severe plaque psoriasis: Results from the ECLIPSE study. J. Dermatol. Treat..

[9] McGonagle D., McInnes I.B., Deodhar A., Schett G., Shawi M., Kafka S., Karyekar C.S., Kollmeier A.P., Hsia E.C., Xu X.L. (2021). Resolution of enthesitis by guselkumab and relationships to disease burden: 1-year results of two phase 3 psoriatic arthritis studies. Rheumatology.

[10] Menter A., Strober B.E., Kaplan D.H., Kivelevitch D., Prater E.F., Stoff B., Armstrong A.W., Connor C., Cordoro K.M., Davis D.M. (2019). Joint AAD-NPF guidelines of care for the management and treatment of psoriasis with biologics. J. Am. Acad. Dermatol..

[11] Ruggiero A., Fabbrocini G., Cinelli E., Ocampo Garza S.S., Camela E., Megna M. (2022). Anti-interleukin-23 for psoriasis in elderly patients: Guselkumab, risankizumab and tildrakizumab in real-world practice. Clin. Exp. Dermatol..

[12] Gisondi P., Fargnoli M.C., Amerio P., Argenziano G., Bardazzi F., Bianchi L., Chiricozzi A., Conti A., Corazza M., Costanzo A. (2022). Italian adaptation of EuroGuiDerm guideline on the systemic treatment of chronic plaque psoriasis. Ital. J. Dermatol. Venereol..

[13] European Medicines Agency (2024). Tremfya (Guselkumab) 100 mg Solution for Injection: Summary of Product Characteristics.

[14] Italian Medicines Agency (AIFA) (2008). Guidelines for the classification and conduct of observational studies on medicines. Official Gazette of the Italian Republic, No. 76.

[15] Reich K., Duffin K.C., Ho V., Tsai T.-F., Puig L., Thaçi D., Papp K.A., Gottlieb A.B., Song M., Miller M. Response to Guselkumab in Patients 65 Years of Age or Older with Moderate to Severe Plaque Psoriasis: Results from VOYAGE 1 and VOYAGE 2 Through Week 28. Proceedings of the 29th European Academy of Dermatology and Venereology (EADV) Virtual Congress.

[16] Singh S., Facciorusso A., Singh A.G., Casteele N.V., Zarrinpar A., Prokop L.J., Grunvald E.L., Curtis J.R., Sandborn W.J. (2018). Obesity and response to anti-tumor necrosis factor-α agents in patients with select immune-mediated inflammatory diseases: A systematic review and meta-analysis. PLoS ONE.

[17] Gargiulo L., Ibba L., Malagoli P., Amoruso F., Argenziano G., Balato A., Bardazzi F., Burlando M., Carrera C.G., Damiani G. (2024). Effectiveness, Tolerability, and Drug Survival of Risankizumab in a Real-World Setting: A Three-Year Retrospective Multicenter Study—IL PSO. J. Clin. Med..

[18] Hagino T., Onda M., Saeki H., Fujimoto E., Kanda N. (2024). Effectiveness of bimekizumab for genital, nail, and scalp lesions with psoriasis: A 24-week real-world study. J. Dermatol..

[19] Bernardini N., Dattola A., Gemma G.P.A., Atzori L., Artosi F., Biondi G., Campione E., Cuccia A., Dessi P., Di Cesare A. (2025). Psoriasis severity, comorbidity burden, and biologic therapy: A multicenter observational study using the Charlson Comorbidity Index. J. Dermatol. Treat..

[20] van Winden M.E.C., van der Schoot L.S., Arias M.v.d.L., van Vugt L.J., Reek J.M.P.A.v.D., van de Kerkhof P.C.M., de Jong E.M.G.J., Lubeek S.F.K. (2020). Effectiveness and Safety of Systemic Therapy for Psoriasis in Older Adults. JAMA Dermatol..

[21] Sandhu V.K., Ighani A., Fleming P., Lynde C.W. (2020). Biologic Treatment in Elderly Patients With Psoriasis: A Systematic Review. J. Cutan. Med. Surg..

[22] Phan C., Beneton N., Delaunay J., Reguiai Z., Boulard C., Fougerousse A., Cinotti E., Romanelli M., Mery-Bossard L., Thomas-Beaulieu D. (2020). Effectiveness and Safety of Anti-interleukin-17 Therapies in Elderly Patients with Psoriasis. Acta Derm. Venereol..

[23] Osuna C.G., García S.R., Martín J.C., Jiménez V.G., López F.V., Santos-Juanes J. (2021). Use of Biological Treatments in Elderly Patients with Skin Psoriasis in the Real World. Life.

[24] Glazer Levavi S., Maman R., Sherman S., Mimouni D., Pavlovsky L. (2025). Systemic Biologic Treatment for Psoriasis in Elderly Patients. J. Clin. Med..

[25] Kwon H.H., Kwon I.H., Youn J.I. (2012). Clinical study of psoriasis occurring over the age of 60° years: Is elderly-onset psoriasis a distinct subtype?. Int. J. Dermatol..

[26] Megna M., Potestio L., Fabbrocini G., Camela E. (2022). Treating psoriasis in the elderly: Biologics and small molecules. Expert Opin. Biol. Ther..

[27] Ter Haar E.L.M., Van den Reek J.M.P.A., Gaarn Du Jardin K., Barbero-Castillo A., De Jong E.M.G.J., Lubeek S.F.K. (2023). Efficacy and Safety of Tildrakizumab in Older Patients: Pooled Analyses of Two Randomized Phase III Clinical Trials (reSURFACE 1 and reSURFACE 2) Through 244 Weeks. Acta Derm. Venereol..

[28] Dattola A., Bernardini N., Caldarola G., Coppola R., De Simone C., Giordano D., Giunta A., Moretta G., Pagnanelli G., Panasiti V. (2024). Effectiveness of Ixekizumab in Elderly Patients for the Treatment of Moderate-to-Severe Psoriasis: Results from a Multicenter, Retrospective Real-Life Study in the Lazio Region. Dermatol. Pract. Concept..

